# Supplementary material for: A long-range cis-regulatory element for class I odorant receptor genes
Source: Nat Commun. 2017 Oct 12;8:885. doi: 10.1038/s41467-017-00870-4 (PMC5638857; doi:10.1038/s41467-017-00870-4)
Supplement: Supplementary file 1 — Supplementary Information [file 41467_2017_870_MOESM1_ESM.pdf]

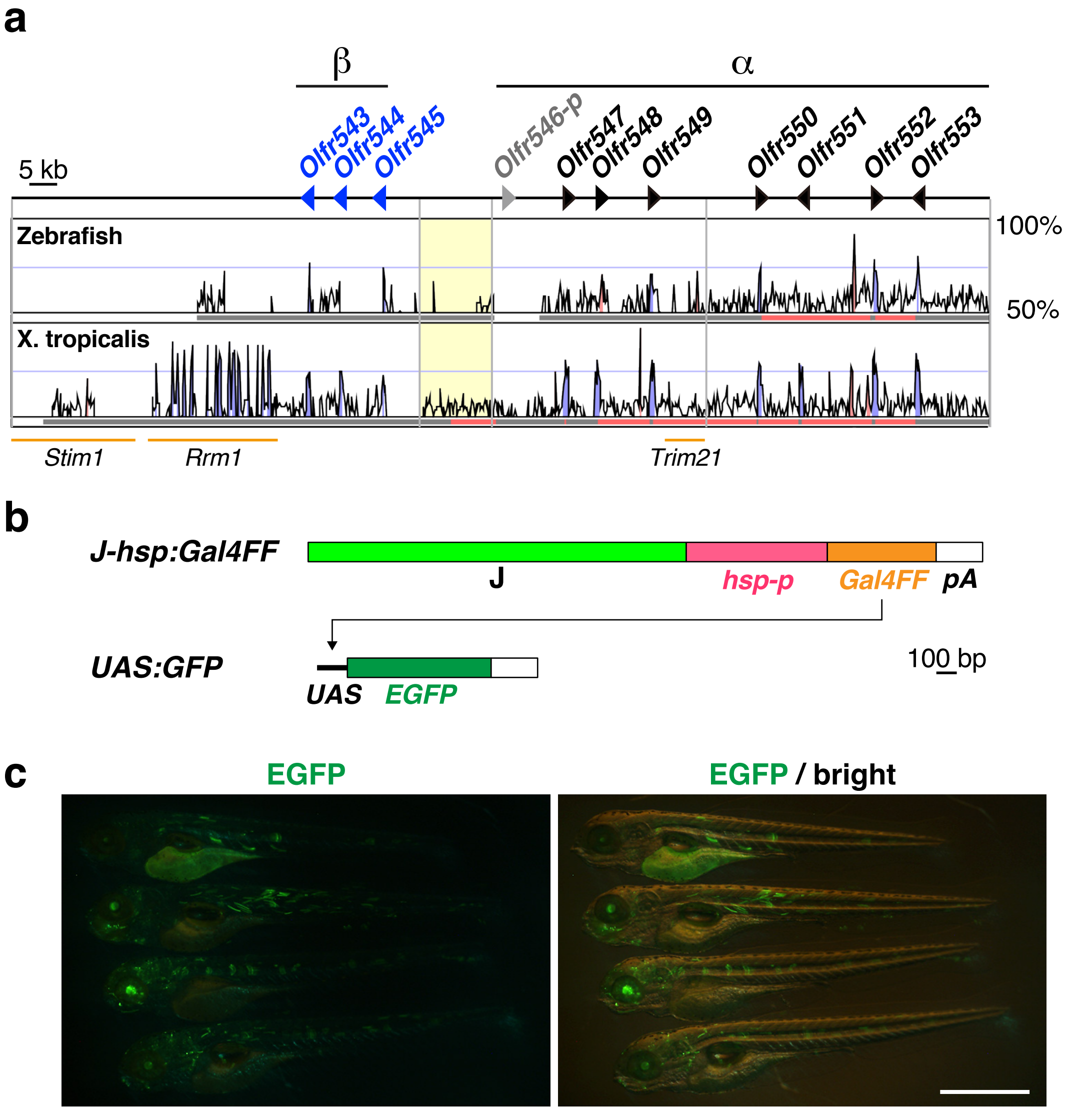

Supplementary Figure 1 | Conservation and functional analyses of the J element in nonmammals.

(a) Genomic region of the *Olfr544* locus (same as Fig. 1b) and nucleotide percent identity plots between mouse and zebrafish or *X. tropicalis* using VISTA. No significant conservation in the intergenic region was detected, even when a 50-bp calculation window was used for high-sensitivity detection.

(b,c) Transient reporter assay of enhancer activity of the J element in zebrafish. **(b)** Strategy of the transient reporter assay in zebrafish. The J element was fused to the *hsp* basal promoter (*hsp-p*), *Gal4FF* (a modified GAL4 transcription activator), and a polyadenylation sequence (*pA*). To test the enhancer function of the J element, the J-hspGal4FF driver construct was injected into *UAS:GFP* zebrafish embryos. **(c)** Whole-mount fluorescent images of injected larvae at 4 dpf (days post-fertilization). Specific GFP expression was not observed in the nose (n = 77), indicating the J element did not show any enhancer activity in the transient reporter assay in zebrafish. The scale bar is 500 μm.

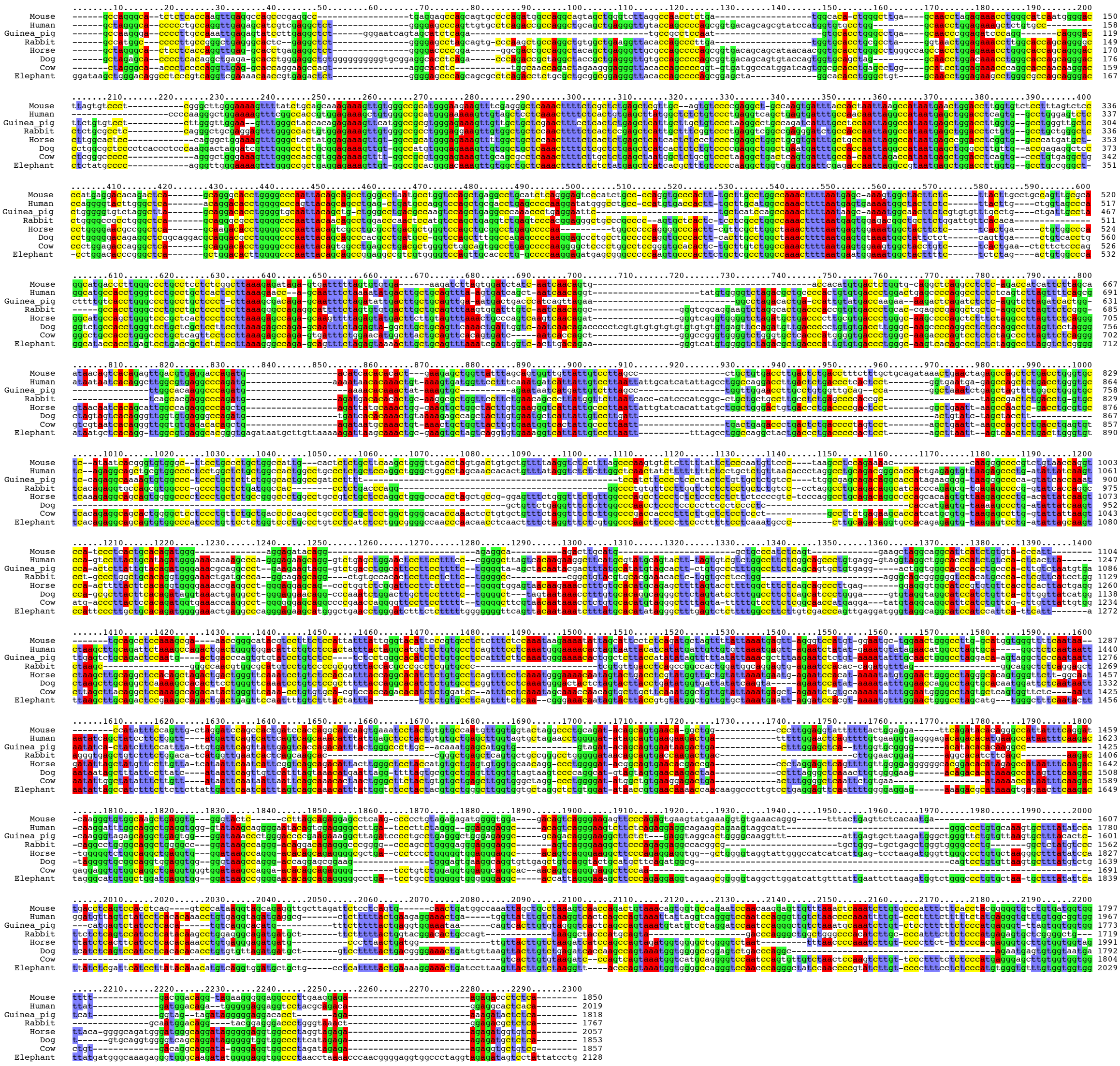

Supplementary Figure 2 | Multiple alignment of the J element.

Multiple alignment of the J element for mouse and 7 representative placental mammalian species, human (Primates), guinea pig (Rodentia), rabbit (Lagomorpha), horse (Perissodactyla), dog (Carnivora), cow (Cetartiodactyla), and African elephant (Proboscidea).

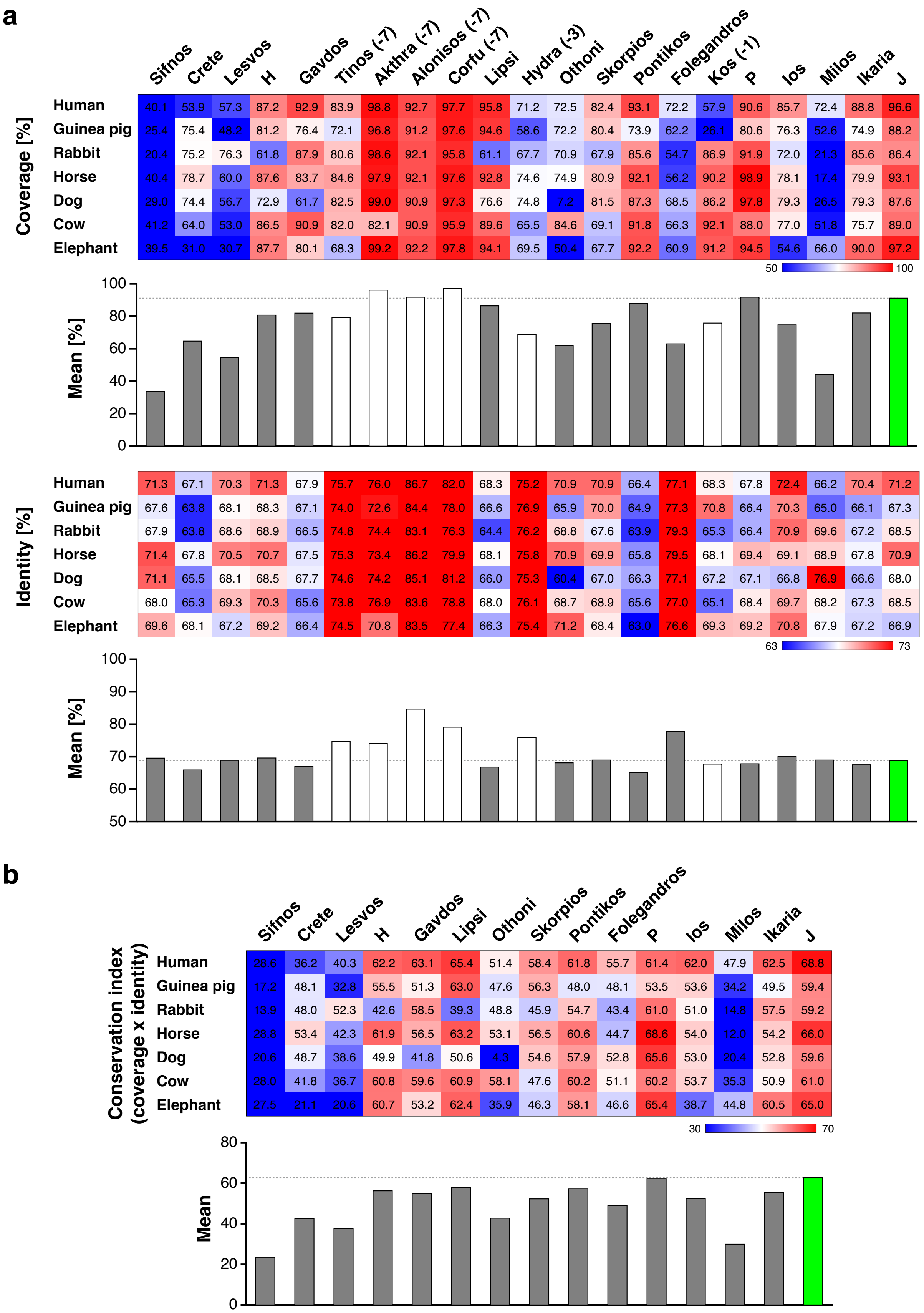
a
Sifnos Crete Lesvos H Gavdos Tinos (-7) Akthra (-7) Alonisos (-7) Corfu (-7) Lipsi Hydra (-3) Othoni Skorpios Pontikos Folegandros Kos (-1) P Ios Milos Ikaria J
Coverage [%]
Human 40.1 53.9 57.3 87.2 92.9 83.9 98.8 92.7 97.7 95.8 71.2 72.5 82.4 93.1 72.2 57.9 90.6 85.7 72.4 88.8 96.6
Guinea pig 25.4 75.4 48.2 81.2 76.4 72.1 96.8 91.2 97.6 94.6 58.6 72.2 80.4 73.9 62.2 26.1 80.6 76.3 52.6 74.9 88.2
Rabbit 20.4 75.2 76.3 61.8 87.9 80.6 98.6 92.1 95.8 61.1 67.7 70.9 67.9 85.6 54.7 86.9 91.9 72.0 21.3 85.6 86.4
Horse 40.4 78.7 60.0 87.6 83.7 84.6 97.9 92.1 97.6 92.8 74.6 74.9 80.9 92.1 56.2 90.2 98.9 78.1 17.4 79.9 93.1
Dog 29.0 74.4 56.7 72.9 61.7 82.5 99.0 90.9 97.3 76.6 74.8 7.2 81.5 87.3 68.5 86.2 97.8 79.3 28.5 79.3 87.6
Cow 41.2 64.0 53.0 86.5 90.9 82.0 82.1 90.9 95.9 89.6 65.5 84.6 69.1 91.8 66.3 92.1 88.0 77.0 51.8 75.7 89.0
Elephant 39.5 31.0 30.7 87.7 80.1 68.3 99.2 92.2 97.8 94.1 69.5 50.4 67.7 92.2 60.9 91.2 94.5 54.6 66.0 90.0 97.2
50 100
Mean [%]
0 20 40 60 80 100
Identity [%]
Human 71.3 67.1 70.3 71.3 67.9 75.7 76.0 86.7 82.0 68.3 75.2 70.9 70.9 66.4 77.1 68.3 67.8 72.4 66.2 70.4 71.2
Guinea pig 67.6 63.8 68.1 68.3 67.1 74.0 72.6 84.4 78.0 66.6 76.9 65.9 70.0 64.9 77.3 70.8 66.4 70.3 65.0 66.1 67.3
Rabbit 67.9 63.8 68.6 68.9 66.5 74.8 74.4 83.1 76.3 64.4 76.2 68.8 67.6 63.9 79.3 65.3 66.4 70.9 69.6 67.2 68.5
Horse 71.4 67.8 70.5 70.7 67.5 75.3 73.4 86.2 79.9 68.1 75.8 70.9 69.9 65.8 79.5 68.1 69.4 69.1 68.9 67.8 70.9
Dog 71.1 65.5 68.1 68.5 67.7 74.6 74.2 85.1 81.2 66.0 75.3 60.4 67.0 66.3 77.1 67.2 67.1 66.8 76.9 66.6 68.0
Cow 68.0 65.3 69.3 70.3 65.6 73.8 76.9 83.6 78.8 68.0 76.1 68.7 68.9 65.6 77.0 65.1 68.4 69.7 68.2 67.3 68.5
Elephant 69.6 68.1 67.2 69.2 66.4 74.5 70.8 83.5 77.4 66.3 75.4 71.2 68.4 63.0 76.6 69.3 69.2 70.8 67.9 67.2 66.9
63 73
Mean [%]
50 60 70 80 90 100
b
Sifnos Crete Lesvos H Gavdos Lipsi Othoni Skorpios Pontikos Folegandros P Ios Milos Ikaria J
Conservation index (coverage x identity)
Human 28.6 36.2 40.3 62.2 63.1 65.4 51.4 58.4 61.8 55.7 61.4 62.0 47.9 62.5 68.8
Guinea pig 17.2 48.1 32.8 55.5 51.3 63.0 47.6 56.3 48.0 48.1 53.5 53.6 34.2 49.5 59.4
Rabbit 13.9 48.0 52.3 42.6 58.5 39.3 48.8 45.9 54.7 43.4 61.0 51.0 14.8 57.5 59.2
Horse 28.8 53.4 42.3 61.9 56.5 63.2 53.1 56.5 60.6 44.7 68.6 54.0 12.0 54.2 66.0
Dog 20.6 48.7 38.6 49.9 41.8 50.6 4.3 54.6 57.9 52.8 65.6 53.0 20.4 52.8 59.6
Cow 26.0 41.8 36.7 60.8 59.6 60.9 58.1 47.6 60.2 51.1 60.2 53.7 35.3 50.9 61.0
Elephant 27.5 21.1 20.6 60.7 53.2 62.4 35.9 46.3 58.1 46.6 65.4 38.7 44.8 60.5 65.0
30 70
Mean
0 20 40 60 80

Supplementary Figure 3 | Conservation analysis of the mouse OR enhancers conserved in all tested mammals.

(a) The J element and 20 of 35 previously identified OR enhancers were conserved in all seven tested placental mammalian genomes. Coverage of alignment region (upper part) and nucleotide identity (lower part) of the mouse OR enhancers for other mammalian species are summarized. The graph shows the mean for the seven species. Sifnos, Crete, Lesvos, H, Gavdos, Lipsi, Othoni, Skorpios, Pontikos, Folegandros, P, Ios, Milos, Ikaria, and J elements are conserved and are located in proximity to OR genes in all tested placental mammalian genomes (J, green bar; the others, grey bars in graph). However, Tinos, Akthra, Alonisos, Corfu, Hydra, and Kos do not reside close to OR genes in at least one non-mouse genomes (white bars in graph). The number of genomes containing enhancers that do not reside close to the OR gene is shown in parentheses. The bottom graph shows the mean among the seven species.

(b) Summary of the conservation analysis for each enhancer-species combination of the selected 15 elements, which are all conserved in the seven tested mammals and linked to OR genes. The conservation index is defined by multiplying the percentage coverage by the nucleotide identity. Nine elements (H, Gavdos, Lipsi, Skorpios, Pontikos, P, Ios, Ikaria, and J) showed relatively high average conservation indexes (mean > 50). The J element was one of the most highly conserved of the 15 conserved OR enhancer elements.

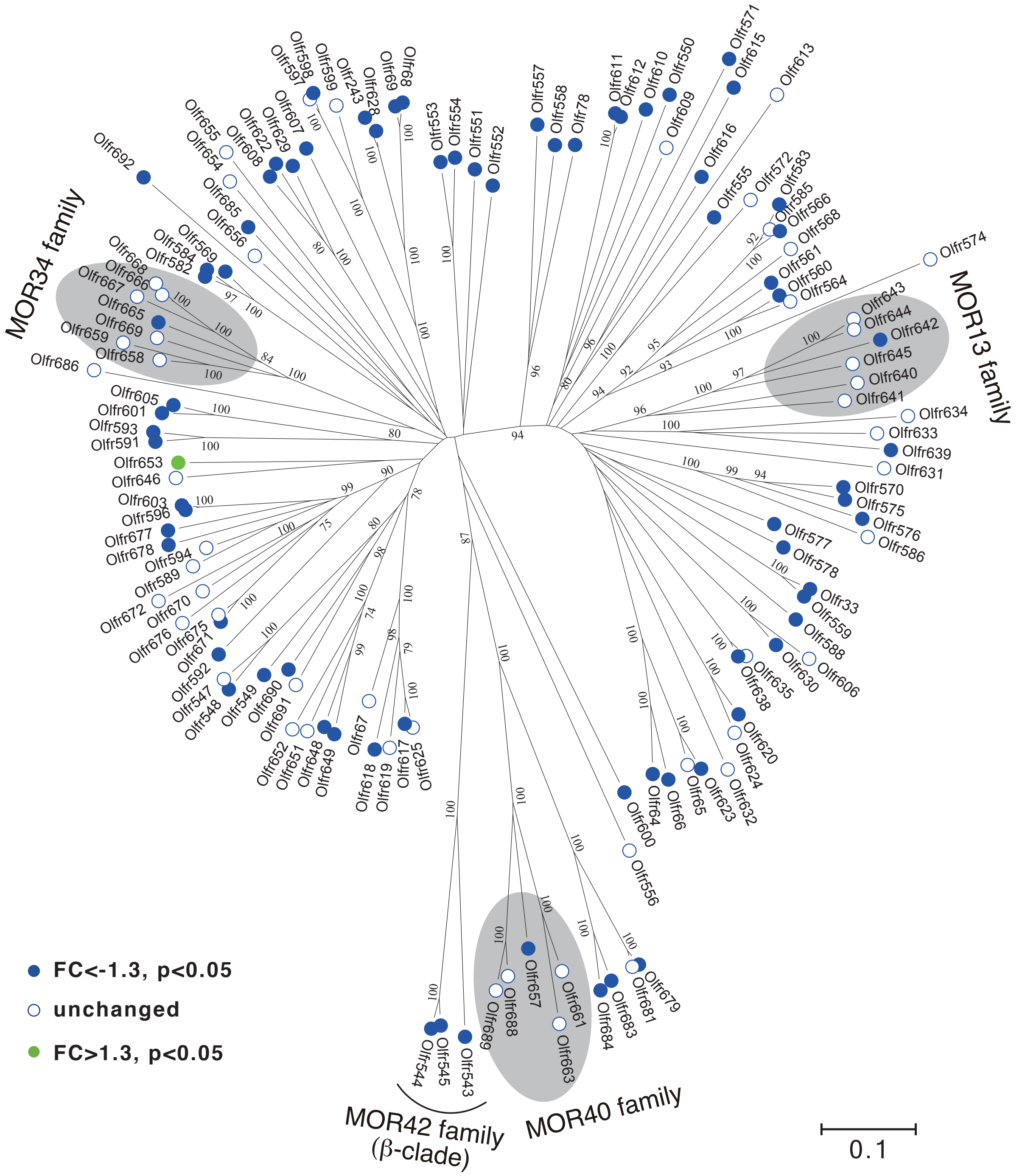

Supplementary Figure 4 | Phylogenetic analysis of the effect of ΔJ on the expression of class I OR genes.

An unrooted phylogenetic tree of the mouse class I OR family using 129 intact class I OR genes was constructed by the neighbor-joining method. Class I OR family is classified into 42 subfamilies. Differentially expressed genes (1.3-fold change, $p < 0.05$) are indicated by filled circles (downregulated, blue; upregulated, green), whereas unaffected genes are represented by open circles. The class I genes whose expression levels were affected by ΔJ were not biased against specific subfamilies; however, the MOR13, MOR34, and MOR40 subfamilies included many J-independent genes (grey shaded). Bootstrap values obtained from 500 resamplings are shown only for nodes with bootstrap values > 70%.

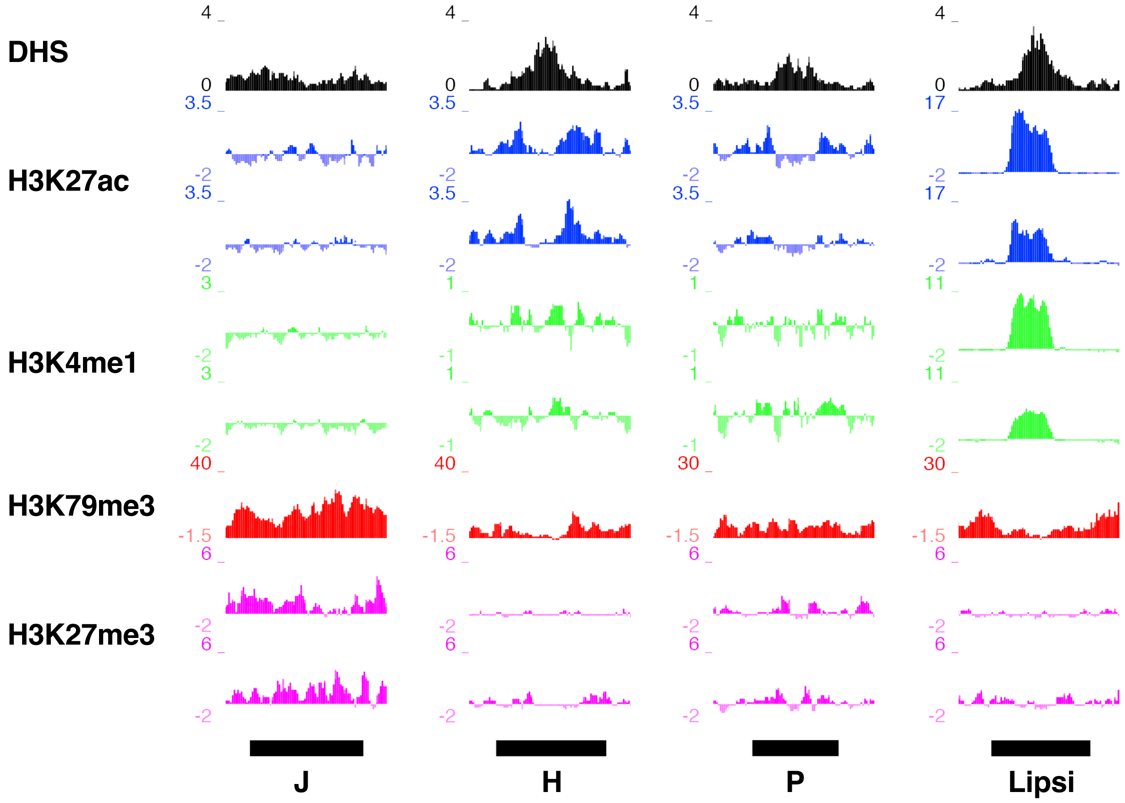

Supplementary Figure 5 | An epigenetic signature for the J and class II enhancer elements. Sequencing tracks for DNaseI hypersensitivity (DHS) sequencing and chromatin immunoprecipitation sequencing on the J and class II enhancer element regions (H, P, and Lipsi). Sequencing data were retrieved from GSE55174 and GSE52464 (single data for DHS and H3K79me3; two replicates for H3K27ac, H3K4me1, and H3K27me3) deposited in Gene Expression Omnibus. Note these sequence data were obtained from whole MOE preparations. Class II enhancer elements have a well-defined DHS peak, are enriched for H3K27ac and H3K4me1, and are missing H3K79me3 and H3K27me3 modifications. In contrast, the J element does not have significant DHS, H3K27ac and H3K4me1 peaks, and is enriched for H3K79me3 and H3K27me3, indicating that epigenetic landscape of the J element is different from previously characterized enhancer elements for class II genes.

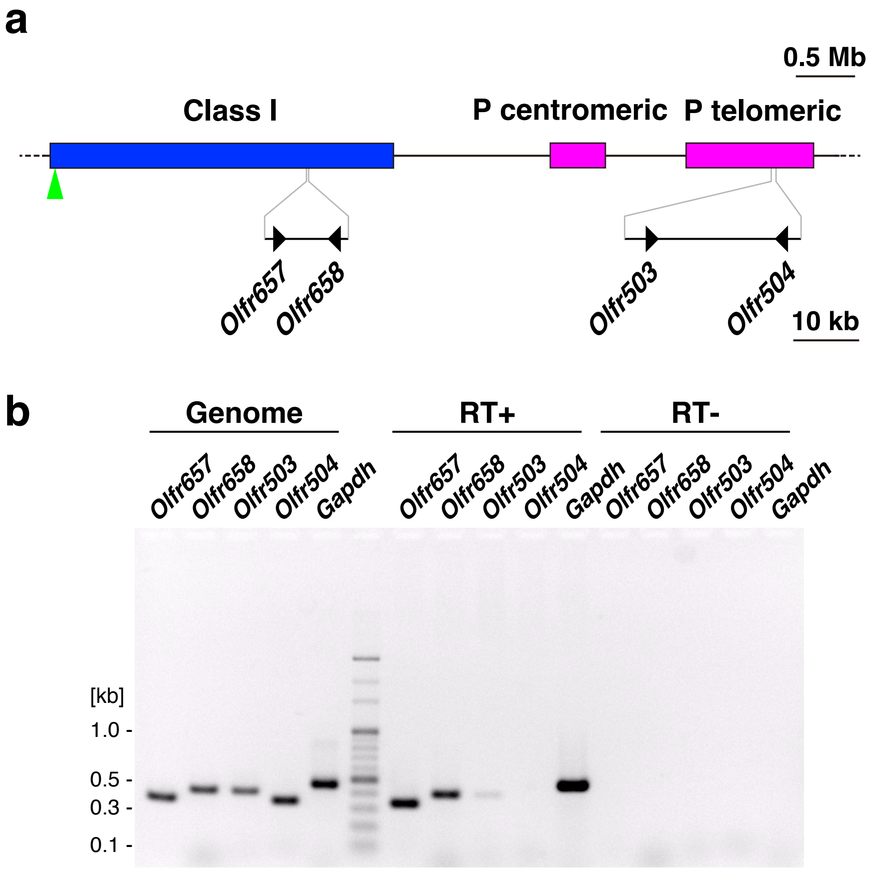

Supplementary Figure 6 | Expression analysis of *Olfr503* and *Olfr504*.

(a) Genomic organization of *Olfr503* and *Olfr504* and the homologous genes *Olfr657* and *Olfr658*. *Olfr657* and *Olfr658* reside in the 3-Mb class I OR cluster; *Olfr503* and *Olfr504* are located in the P telomeric cluster. The green triangle represents the J element.

(b) RT-PCR analysis for the four class I OR genes and *Gapdh* on RT+ (with reverse transcriptase) or RT- (without reverse transcriptase) cDNA derived from wild-type MOE. Genomic DNA was used as a positive control. The expression of *Olfr657* and *Olfr658* is clearly detected, whereas *Olfr503* and *Olfr504* expression is not detected or is barely detected.

Supplementary Table 1 | ISH analysis in ΔJ mice.

OR gene probe	n	Expression [% of WT] (mean ± SEM)	Microarray analysis fold change value
**Class I gene**			
Olfr543	3	16 ± 6.4	-1.61
Olfr544	5	2.1 ± 0.5	-3.57
Olfr547	3	50 ± 12	-1.03
Olfr552	4	4.6 ± 1.6	-6.36
Olfr33	5	35 ± 3.7	-2.29
Olfr584	4	72 ± 21	-1.70
Olfr607	4	26 ± 4.4	-3.14
Olfr639	3	19 ± 2.5	-3.28
Olfr653	5	159 ± 13	1.56
Olfr657	4	3.9 ± 1.6	-3.37
Olfr690	4	56 ± 7.9	-1.58
Olfr692	5	29 ± 4.9	-1.39
**Atypical class I gene**			
Olfr556	3	76 ± 4.5	-1.05
Olfr686	3	78 ± 14	-1.08
**Class II gene (dorsal)**			
Olfr151	3	106 ± 21	-1.05
Olfr521	5	109 ± 17	-1.06
**Class II gene (ventral)**			
Olfr17	4	97 ± 9.1	-1.10
Olfr693	4	100 ± 20	-1.04

Summary of ISH analysis for 18 tested OR genes (12 class I, 2 atypical class I and 4 class II genes). Percentage of expression was calculated by dividing the average number per section of ΔJ by that of wild-type in each experiment pair. The fold change value of the microarray data (Supplementary Data 2) was displayed on the right column (ratio of signals between ΔJ and wild-type mice; positive value means ΔJ per wild-type, negative value means wild-type per ΔJ).

Supplementary Table 2 I ISH analysis in ΔJ heterozygous and homozygous mice.

OR gene probe	Expression [% of WT] J/J		J/ΔJ		ΔJ/ΔJ		One-way ANOVA p-value
	n	(mean ± SEM)	n	(mean ± SEM)	n	(mean ± SEM)	
Olfr544	5	100 ± 13	4	75 ± 7.6	5	1.9 ± 0.3	< 0.0001
Olfr552	4	100 ± 7.7	4	66 ± 16	4	4.9 ± 1.9	0.0004
Olfr33	5	100 ± 14	4	75 ± 9.2	5	34 ± 3.3	0.0014
Olfr657	4	100 ± 12	4	80 ± 11	4	3.5 ± 1.3	0.0001
Olfr692	5	100 ± 13	4	90 ± 10	5	29 ± 6.4	0.0008
Olfr653	5	100 ± 16	4	93 ± 5.1	4	179 ± 29	0.0178

Summary of ISH analysis for 6 tested class I OR genes. Percentage of expression was calculated by dividing the average number per section of ΔJ heterozygous (J/ΔJ) or homozygous (ΔJ/ΔJ) by that of wild-type (J/J). P values were calculated by the One-way ANOVA in each *Olfr* probes.

Supplementary Table 3 I List of PCR primers.

Target	Forward primer sequence	Reverse primer sequence	Product size [bp]
**Homology arm for deletion of Tg-220**			
Tg-U79	CCCAAGCTTATCACCAGGGACAACTTGG	CGGGATCCAGAGGTGCAAGAAACTGAC	1201
Tg-U29	GGCCATTCAAGCTTCTACTCTCTAC	CGGGATCCACTTCCATGACCTGAGTC	1260
Tg-U16	CTCAAGCTTGTCTGCCCTCTCTGAC	CGGGATCCTTGACGGTGGCTCTGATTG	1223
**Reporter transgene**			
Olfr544 promoter	GCTCTAGAGCACACCCCTTCAGCTCCTC	TCCCCGCGGCAGGACTCACACAGGCAGGG	921
5´-end of 13 kb region	ACGCGTCGACTCAGTATCAGGCCATTC	CGGGATCCTAGTTCAGCTGTCGCTTTCC	2414
3´-end of 13 kb region	CGGGATCCATAGATCCGCCTACTTCCAC	GCTCTAGAGAGGGCTCAACATTGCTTTG	876
**Zebrafish transgene**			
1.9 kb J element	CCGCTCGAGTAGTTTTCTGTGAGAGGGTCTCTTC	CTAGTCTAGAGGGGGACAAAGCCAGGGCAT	1894
**Mouse genotyping**			
J (intact) allele	GCTTAGATTCTCCTCAGTGCAAC	CACTTGCCTGCTACTGTCATTC	432
ΔJ allele	ACTGGCATCATCTCTACATCAGC	CACTTGCCTGCTACTGTCATTC	299 for ΔJ line#8 (2281 for intact allele)
Venus	GCAAGCTGACCCTGAAGCTG	TTGCTCAGGGCGGACTGGTA	508
GFP	GGCATCAAGGTGAACTTCAAGATCC	CTTTACTTGTACAGCTCGTCCATGC	242
SacB	GCTGAATACAACGGCTATCACG	TCTCTCAGCGTATGGTTGTCG	449
CmR	GAGGCATTTCAGTCAGTTGCTC	CGGCATGATGAACCTGAATCG	517
**RT-PCR**			
Olfr657	CCTTCAATAGTTCCAAGTCCCA	AAGATACCGGACTCCATGCC	343
Olfr658	GTCATGTCAGTGCAGAATAGCA	AGTAACGCAGAGGGTAGCAG	406
Olfr503	TCATGTCAGCGAGGAATAACC	GAGTAATGCAGAGGGTAGCAA	406
Olfr504	AGACTTCAATAGTTCCAAGTTCCT	GCAGATACCAGACTCCATGCT	346
Gapdh	ACCACAGTCCATGCCATCAC	ATGTAGGCCATGAGGTCCAC	467

Supplementary Table 4 l Off-target analysis for ΔJ mice.

Target	Chr	Seed sequence (20-bp + PAM)	Forward primer sequence	Reverse primer sequence	Mutation
gRNA1 target	7	tctcattgccacccggatga**tgg**	-	-	-
OT-1	10	cactgctgccacccggatga**agg**	tttgtacttgagctggtgaggc	tgtctcttgccaccacactg	No
gRNA2 target	7	gcccctccaccgtacttgca**ggg**	-	-	-
No potential off-target sites					

Supplementary Table 5 I List of riboprobes.

Gene	Synonym	OR class	Accession #	Nucleotide position	Cross-hybridized gene	Reference
**OR coding probe**						
Olfr543	MOR42-2	I	NM_001011782	38-987	-	
Olfr544	MOR42-3, S6	I	NM_020289	14-915	Olfr545	
Olfr547	MOR31-4	I	NM_147079	31-910	-	
Olfr552	MOR28-1	I	NM_147102	15-927	-	
Olfr556	MOR41-1	atypical I	NM_146754	82-994	-	
Olfr33	MOR11-2	I	NM_147073	1-957	Olfr559	
Olfr78	MOR18-2, MOL2.3	I	NM_130866	1140-2102	-	
Olfr578	MOR7-1	I	NM_147115	4-790	-	
Olfr584	MOR30-2	I	NM_147054	71-748	Olfr569, Olfr582	
Olfr607	MOR33-3P	I	XM_006544227	1-843	-	
Olfr639	MOR12-1	I	NM_147084	1-922	-	
Olfr653	MOR33-1	I	NM_147074	32-960	-	
Olfr657	MOR40-13	I	NM_146312	1-913	Olfr504	
Olfr672	MOR32-4	I	NM_146760	9-919	-	
Olfr686	MOR35-1	atypical I	NM_147069	39-948	-	
Olfr690	MOR31-2, Ors18	I	NM_020290	8-1002	-	
Olfr692	MOR36-1	I	NM_146355	23-952	-	
Olfr521	MOR101-2	II	NM_146356	19-963	Olfr520	
Olfr693	MOR283-8	II	NM_146453	13-928	-	
Olfr54	MOR126-1	II	NM_010997	31-933	Olfr1375	
Olfr73	MOR174-9, mOR-EG	II	NM_054090	3-904	-	
Olfr878	MOR163-1	II	NM_146798	233-909	-	
Olfr17	MOR263-5, P2	II	AF247657	54–948	-	Fuss et al., (2007)
Olfr151	MOR171-2, M71	II	AF281061	64-930	Olfr160	Fuss et al., (2007)
Olfr19	MOR140-1, M12	II	AY073758	172–869	-	Fuss et al., (2007)
**Olfr545 specific probe**						
Olfr545 3'UTR	MOR42-1, S50	I	NC_000073.6	102492367-102493333	-	
**Others**						
GFP	-	-	U55762	677-1416	Venus	
