# Supplementary material for: A long-range cis-regulatory element for class I odorant receptor genes
Source: Nat Commun. 2017 Oct 12;8:885. doi: 10.1038/s41467-017-00870-4 (PMC5638857; doi:10.1038/s41467-017-00870-4)
Supplement: Supplementary file 3 — Supplementary Files [file 41467_2017_870_MOESM3_ESM.pdf]

Description of Additional Supplementary Files

File Name: Supplementary Data 1
Description: **List of the conserved 21 OR enhancer elements and nearest OR genes in 8 placental mammalian species.** Genomic information for the 21 conserved OR enhancer elements and their nearest OR genes was summarized. Eight placental mammalian species examined and their genomic data are mouse (mm9), human (hg19), guinea pig (cavPor3), rabbit (oryCun2), horse (equCab2), dog (canFam3), caw (bosTau8), and African elephant (loxAfr3). "X" in nearest OR gene column means an element has no OR gene within a 1-Mb region on the same chromosome or on the same scaffold. Names of some OR genes, which have not been named because of updated genome data, are shown by "-".

File Name: Supplementary Data 2
Description: **Microarray data of odorant receptor genes.** Analyzed microarray data for 1213 *Olfr* gene probe sets from six ΔJ and six wild-type mice. Correlated p values were calculated by the moderated *t*-test with multiple testing correction of Benjamini Houchberg FDR.
